# Long-acting olanzapine versus long-acting risperidone for schizophrenia in Spain – a cost-effectiveness comparison

**DOI:** 10.1186/s12888-014-0298-4

**Published:** 2014-12-02

**Authors:** Tatiana Dilla, Jörgen Möller, Paul O’Donohoe, María Álvarez, José A Sacristán, Michael Happich, Antje Tockhorn

**Affiliations:** Eli Lilly Spain, Av. de la Industria, 30, 28108 Alcobendas, Madrid Spain; Evidera, 1 Butterwick, Hammersmith, London, W6 8DL UK; CRF Health, 229–243 Brook House, Shepherds Bush Road, London, W6 7AN UK; Eli Lilly Deutschland GmbH, Werner-Reimers-Strasse 2-4, 61352 Bad Homburg, Germany; Eli Lilly UK, Erl Wood Manor, Sunninghill Road, Windlesham, Surrey, GU20 6PH UK

**Keywords:** Cost-effectiveness analysis, Discrete event simulation, Economic model, Long-acting olanzapine, Long-acting risperidone, Antipsychotic, Schizophrenia, Spain

## Abstract

**Background:**

In schizophrenia, medication adherence is critical to achieve better patient outcomes and to avoid relapses, which are responsible for a significant proportion of total healthcare costs for this chronic illness. The aim of this study was to assess the cost-effectiveness of olanzapine long-acting injection (OLAI) compared with risperidone long-acting injection (RLAI) in patients with schizophrenia in Spain.

**Methods:**

A discrete event simulation (DES) model was developed from a Spanish healthcare system perspective to estimate clinical and economic outcomes for patients with schizophrenia over a five-year period. Patients who had earlier responded to oral medication and have a history of relapse due to adherence problems were considered. Identical model populations were treated with either OLAI or RLAI. In the absence of a head-to-head clinical trial, discontinuation and relapse rates were obtained from open-label studies. The model accounted for age, gender, risks of relapse and discontinuation, relapse management, hospitalization, treatment switching and adverse events. Direct medical costs for the year 2011 and outcomes including relapse avoided, life years (LYs), and quality-adjusted life years (QALYs) were discounted at a rate of 3%.

**Results:**

When comparing RLAI and OLAI, the model predicts that OLAI would decrease 5-year costs by €2,940 (Standard Deviation between replications 300.83), and result in a QALY and LY gains of 0.07 (SD 0.019) and 0.04 (SD 0.025), respectively. Patients on OLAI had fewer relapses compared to RLAI (1.392 [SD 0.035] vs. 1.815 [SD 0.035]) and fewer discontinuations (1.222 [SD 0.031] vs. 1.710 [SD 0.039]). Sensitivity analysis indicated that the study was robust and conclusions were largely unaffected by changes in a wide range of parameters.

**Conclusions:**

The present evaluation results in OLAI being dominant over RLAI, meaning that OLAI represents a more effective and less costly alternative compared to RLAI in the treatment of patients with schizophrenia in the Spanish setting.

## Background

Schizophrenia is a chronic illness associated with considerable clinical, social, and economic consequences. Schizophrenia results in enduring symptoms and prolonged functional impairment. Estimates of the prevalence of schizophrenia in Spain, calculated from epidemiological and demographic data, indicated a mean prevalence of 3.0 per 1000 inhabitants per year for men and 2.86 per 1000 for women per year [1]. Suboptimal adherence to prescribed treatment is a prime driver of relapses, rehospitalisation, and persistence of psychotic symptoms. Noncompliance rates vary from 41.2 to 49.5% in patients with schizophrenia [2,3].

Schizophrenia is an extremely resource-intensive disorder. The costs of relapses and rehospitalisation have significant effects on healthcare budgets [4-7]. Direct costs data from Spain indicate that healthcare expenditure associated with schizophrenia accounts for about 2.7% of total public healthcare expenditure in 2002 [7]. The total costs of schizophrenia in 2002 in Spain were estimated at €1,971 million with direct medical costs (hospitalisation, outpatients consultation and drug costs) contributing significantly (53%) to the total cost [7,8]. The Schizophrenia Outpatient Health Outcomes (SOHO) study, using unit costs based on UK Department of Health data and inflated to 2005 prices showed that the cost of managing relapse in patients with schizophrenia is about £14,055 [9]. Several studies confirm that schizophrenia imposes a high burden on national health-care costs [10-13].

Pharmacological management of schizophrenia relies greatly on antipsychotic drugs. Olanzapine pamoate monohydrate is a long-acting intramuscular depot formulation (OLAI) indicated for the maintenance treatment of adult patients with schizophrenia sufficiently stabilised during acute treatment with oral olanzapine [14]. Risperidone long-acting injection (RLAI) is a combination of extended release microspheres for injection and diluent for parenteral use; it is indicated for the maintenance treatment of schizophrenia in patients currently stabilised with oral antipsychotics [15].

Long-acting injectable antipsychotics may be used as an alternative to oral medication therapy in the case of patients with schizophrenia for whom adherence is a clinically significant problem [16].

The general safety and tolerability profile for OLAI is similar to that for oral olanzapine [17]. However, during the pre-marketing clinical trial program, a small proportion of OLAI recipients experienced signs and symptoms consistent with olanzapine overdose described as “post-injection syndrome”. The clinical features of post-injection syndrome included sedation, and/or delirium [18,19]. It has been suggested that the syndrome may be a result of unintended partial intravascular injection or blood vessel injury during the injection [18,19]. This syndrome occurred in <0.1% of injections and approximately 2% of patients [14]. According to the OLAI (Zypadhera®) SPC, an observation period of 3 hours is required following administration of OLAI [14].

The rising cost of health care has increased the need to demonstrate that health care interventions are not only clinically effective, but are also cost-effective. In terms of cost of therapy, psychotic disorders, especially schizophrenia, are considered the most expensive in terms of costs of care per patient [20]. A large part of the cost of managing patients with schizophrenia, based on cost of illness data, stems from short-term hospitalizations and long-term institutional or sheltered living care [20]. Cost data from regulatory clinical trials are generally limited because of the strict modifications made in the practice environment so that the resulting resource use does not reflect the reality of the real world [21]. Economic models can be used to test a wide range of scenarios and strategies to identify the most efficient and equitable allocation of resources [21]. Amongst various economic models, the discrete event simulation model provides a more natural way to simulate clinical reality although requiring more data. The natural time-dependent characteristics of schizophrenia and its progression makes it suitable for using a DES model because it is flexible and represents multiple factors simultaneously [22,23]. Earlier studies using the discrete event simulation (DES) economic model concluded that treating schizophrenia with an atypical antipsychotic agent is cost-effective compared with conventional antipsychotics and is associated with improved quality-adjusted health over a 5-year period [24]. Using a 1-year micro-simulation economic decision model that evaluated the cost-effectiveness of OLAI compared with alternative antipsychotic agents it was found that OLAI is a cost-effective alternative to oral olanzapine and long-acting injection formulations of risperidone and other antipsychotics for the treatment of non-adherent and partially adherent patients with schizophrenia within the United States healthcare system [25].

With a view to determine the cost-effectiveness of OLAI within the Spanish healthcare environment, the present evaluation compares the cost-effectiveness of OLAI versus RLAI. A DES model was developed using relapse and discontinuation rates obtained from indirect comparison of clinical trials and published literature within the perspective of the Spanish healthcare system. The DES model was designed to compare OLAI with the commercially available long-acting second-generation injectable antipsychotic, RLAI, in the management of patients with schizophrenia.

## Methods

Decision trees and Markov models have been widely used in pharmacoeconomic evaluations [26,27]. However, as with all methodologies, these models have many limitations, inherent but inadequate assumptions, and lack the flexibility required to represent appropriately clinical reality [27]. To overcome these limitations in order to accurately capture all of the relevant interdependencies of this chronic, highly heterogeneous disease with limited long-term follow-up data [24] as well as having access to the patient’s differences, individual memory and history, the method of choice was discrete event simulation (DES). The DES model exhibits flexibility in handling perspectives and structural variations with few restrictions. The model closely replicates the natural course of the disease by incorporating multiple factors simultaneously and thus simulating the real-world patient management environment [22-24].

### The model structure

**Figure 1:**
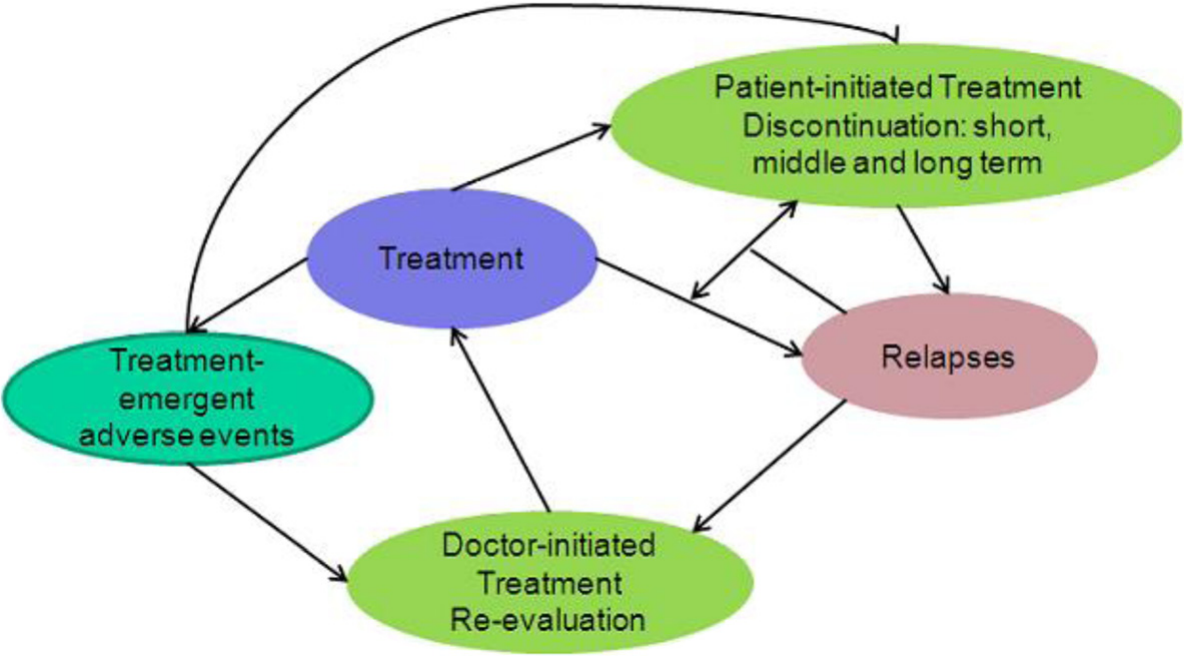
**Factors influencing the Discrete-Event-Simulation model structure.** NB: Although not diagramatically depicted, the model includes the possibility of death from natural causes.

The DES economic model is a patient-level simulation model based on the ARENA® simulation software (Rockwell Automation, Wexford, Pennsylvania, USA). Data entries were made using Microsoft® Excel (Microsoft Corporation, Redmond, Washington, USA). Data were automatically transferred to the ARENA® model. The model generated outputs and results in Microsoft® Excel. The DES model was designed for the present analysis to evaluate the cost-effectiveness of OLAI versus RLAI. It was created around the central theme of relapse and factors influencing the event, with treatment discontinuation regarded as key in the present economic evaluation (Figure 1). The rate of hospitalisation was adopted as a proxy for relapse rates and assumed that all patients entering the model started with zero relapses. Based on attributes of age, gender and background life expectancy, the model created a population of patients who were thereafter cloned (copied) to make it possible to have identical patients in both treatment arms which reduces nuisance variance in the outcomes.

**Figure 2:**
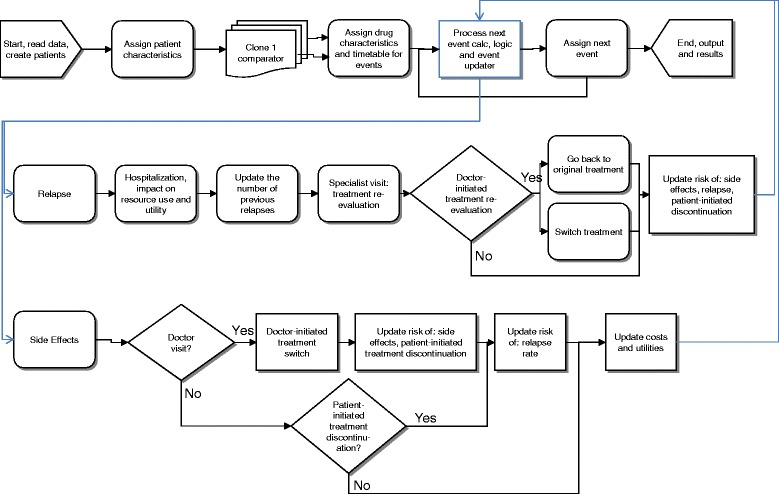
**Flowchart of the discrete-event-simulation model.**

Chronologically, the model recorded all events experienced by patients. These included events such as drug administration, adverse events, remissions, relapses, treatment discontinuations, or death, with costs, life years and benefits being accumulated. When a patient relapsed and was hospitalised, the physician had the choice of continuing the same treatment or switching to another. Adverse events created an opportunity for the patient to initiate a doctor visit for evaluation and treatment. Patients not opting to initiate a doctor visit due to an adverse event remained on the same drug but had the potential for discontinuing from the drug. The model had the ability to differentiate between suicide (which can only happen off-treatment) and background mortality. Figure 2 provides a flowchart describing the discrete-event simulation model build for the comparison of OLAI vs. RLAI.

### The model setting

The time horizon for the model is 5 years. A discount rate of 3% was adopted for both costs and benefits in accordance with recommendations for the Spanish setting [28]. Patient characteristics were taken from patients recruited to the UK SCAP, an observational study with two years of follow up [29]. In the case of patients assigned to OLAI, the model assigned for costs associated with observing patients in the post-injection period based on a nurse visit plus 3 hours of observation time for a group of 8 patients in accordance with an expert panel. Model inputs and sources of information are summarised in Table 1.

**Table 1:** **Resource and costing: data sources (updated to 2011 prices)**

**Data**	**Cost (€)**	**Source**
Annualised cost of outpatient care (based on Spanish data from 2002)	2058.60	Bobes et al., 2004 [30]
Inpatient cost of relapse management based on 2000 Spanish data	4226.91	Peiró et al., [31]
Outpatient cost of relapse management based on 2000 Spanish data (42.94% of inpatient cost)	884.38	Peiró et al., [31]
Cost of switching treatment (assumed at three times the routine cost of schizophrenia management = 3 × €5.64)	16.92/day	Bobes et al., 2004 [30]
		NICE (2009) [32]
Cost associated with post-injection syndrome management, assuming 3 days of additional inpatient hospitalisation (€157.00), one additional day in an emergency psychiatric ward (€107.90) and one additional visit to a psychiatrist (€62.10) = €327.00 based on 2005 costs	368.53	Bernardo et al., 2006 [33]
Cost of administration: OLAI, based on a nurse visit cost of €24.56 plus 3 hours observation time for a group of 8 patients at €10.21	34.77	http://www.oblikue.com/bddcostes/
Cost of administration: RLAI based on a nurse visit cost of €24.56	24.56	http://www.oblikue.com/bddcostes/

The model assumed that the appropriate maintenance dose of OLAI would correspond to oral doses of olanzapine 10 mg/day (the World Health Organization (WHO) defined daily dose for OLZ is 10 mg [34]). In order to achieve this maintenance dose (equivalent to 300 mg/4 weeks), the corresponding starting dose for OLAI is 405 mg/4 weeks (Table 2). Because oral supplementation is not needed with OLAI, the model did not include oral OLZ in the model. The model assumption for RLAI dose was 37.5 mg administered every 14 days (the World Health Organization (WHO) defined daily dose for risperidone depot is 2.7 mg [35]). The suggested starting dose for RLAI is 25 mg/2 weeks (the lowest effective dose) increasing to a maximum (in-label dose) of 50 mg/2 weeks. The 37.5 mg/2 week dose is based on the mid-range dose (Table 2). Consistent with RLAI SPC, patients starting on RLAI receive 21 days of oral risperidone supplementation [RLAI SPC] at an average dose of 2.0 mg/day, according to expert opinion and clinical practice [15,36]. Scheduled administration visits matched the dosage schedule for OLAI and RLAI.

**Table 2:** **Dose and schedule of drug administration**

**Treatment**	**Dose and frequency of administration**
Olanzapine long-acting injection (OLAI)	Starting dose: 405 mg every 4 weeks (cost per day of treatment = €10.69).
	Maintenance dose 300 mg every 4 weeks (cost per day of treatment = €8.32).
Risperidone long-acting injection (RLAI)	37.5 mg every 14 days. Cost per day of treatment = €8.91
	For the first 21 days, patients received additional generic oral risperidone at an average dose of 2.0 mg/day at a cost of €0.103/mg

Cost derived from Colleges of Pharmacists Spanish Council. [Official Medicines Catalog]. 2010; https://botplusweb.portalfarma.com/. Accessed April, 2011. Drug costs expressed in ex-factory price +4% VAT.

### Costs and outcome measures

The model includes the costs associated with treatment and concomitant medications, routine management and follow-up, hospitalisations, switching treatment, and relapse. Outcomes following treatment with OLAI or RLAI or discontinuation of treatment were captured by the model. These included clinical data, cost details, treatment discontinuation rates, relapses, QALYs, LYs, adverse events, death, utilisation of healthcare resources, and direct costs. The model used hospitalisation as a surrogate for relapse. Concomitant medications used by patients generally included anti-depressants, anxiolytics/hypnotics, mood stabilisers, and anticholinergics (Table 3).

**Table 3:** **Concomitant medications**

**Concomitant medications**	**Cost per day**	**Source**
Anti-depressants	€ 0.65	Identified during clinical advisory panels for the SOHO study and are considered to be the most used for patients with schizophrenia
Anxiolytics/Hypnotics	€ 0.10	
Mood stabilisers	€ 0.62	
Anticholinergics	€ 0.07	

Cost derived from Colleges of Pharmacists Spanish Council. [Official Medicines Catalog]. 2010; https://botplusweb.portalfarma.com/. Accessed April, 2011.

Drug costs expressed in ex-factory price +4% VAT.

The outputs of the model are the predicted incremental costs (expressed in € per patient), relapses avoided, the effects (expressed in QALYs and LYs per patient), and the incremental cost-effectiveness ratios (ICERs). Costs and benefits were both discounted at 3% annually in accordance with Spanish guidelines [28]. The ICER is defined as the ratio of the change in costs of an intervention to the change in effects of the intervention. It represents the additional cost of one unit of health outcome gained by a treatment versus the next best alternative. In the Spanish healthcare environment, an ICER of €30,000/QALY gained is considered an acceptable threshold of cost effectiveness [37]. Published medical literature and a clinical expert panel were used to develop baseline model assumptions.

### Data sources

The present model utilised several resource and costing data sources (Table 1) as well as a number of clinical and cost-effectiveness studies to inform patient characteristics and data on adverse events, discontinuation rates, and relapses [38-42]. In the absence of a head-to-head clinical trial, pooled discontinuation and relapse rates were obtained from long-term open-label studies weigthed for different sample size [19,38,39,41]. OLAI is given once a month and RLAI twice a month. The estimation of the daily cost of therapy was based on the ex-factory prices +4% VAT in Spain. The cost of therapy included direct costs such as medications (including any concomitant medication used), administration, cost per relapse/hospitalisation, specialist visits, including psychiatric consult (Table 4). The SOHO database [43,44] was used to derive the tariffs for base utility associated with no relapse, the associated tariffs following relapse, and the utility tariffs associated with the occurrence of adverse events (Tables 5 and 6). In the absence of available data, it was assumed that the utility tariffs for sedation or drowsiness and post-injection syndrome are equivalent to that of sexual dysfunction, which has the highest decrement utility tariff. Adverse event rates for olanzapine and risperidone [Table 7] were based on published reports regarding weight gain, extra-pyramidal symptoms, somnolence, sexual dysfunction, tardive dyskinesia, suicide and post-injection syndrome [14,18,19,40,41,45-47]. For the present study, the risk of post-injection syndrome was set at 2% of patients [14,48].

**Table 4:** **Costs of therapy over a 5-year time horizon**

**Cost particulars**	**OLAI (€)**		**RLAI (€)**	
	**Mean**	***SD***	**Mean**	***SD***
Drug cost^a^	16152.18	*71.50*	16676.88	*82.52*
Administration cost	1949.85	*13.21*	2430.71	*21.77*
Cost per relapse/hospitalisation^b^	6860.26	*169.61*	8955.42	*173.35*
Other costs^c^	9047.91	*41.76*	8886.87	*44.14*
Total cost per patient	34010.21	*207.83*	36949.88	*251.45*

(The Mean and Std Dev are outcomes of the model run over 5 years with 100 replications).

^a^Drug cost includes cost of study drug and concomitant medications.

^b^Since the study assumed hospitalisation as proxy for relapse.

^c^Other costs include specialist visits and other medical costs (i.e. routine management, treatment of adverse events and cost of switching).

SD = Standard deviation.

**Table 5:** **Utilities for relapse**

**Definition**	**Mean**	**SD**
Base utility while no relapse	0.77	0.12
Tariff at relapse	−0.18	0.03
Tariff after 6 months	−0.10	0.02
Tariff after 12 months	−0.07	0.01
Tariff after 18 months	−0.07	0.01

*SD* = Standard deviation. The SD is used as input for the PSA.

**Table 6:** **Utilities for adverse events**

**Adverse events**	**Mean**	**Lower CI**	**Upper CI**
Extra-pyramidal symptoms	−0.054	−0.068	−0.040
Tardive dyskinesia	−0.000	−0.023	0.023
Weight gain	−0.003	−0.014	0.007
Sedation or drowsiness	−0.066	−0.076	−0.056
Sexual dysfunction	−0.066	−0.076	−0.056
Post-injection syndrome	−0.066	−0.076	−0.056

*CI* = Confidence interval. The CI is used as input for the PSA.

**Table 7:** **Adverse event rates (%)**

**Adverse events** ^**a**^	**OLAI**	**RLAI**
Weight gain	9.0	6.0
Extra-pyramidal symptoms	15.0	25.0
Tardive dyskinesia	0.0	0.0
Somnolence	7.0	5.0
Sexual dysfunction	3.0	3.0
Post-injection syndrome	2.0	0.0
Suicide	0.0	0.0

^a^Adverse event rates were taken from [14,40,48].

### Statistics

The base case analysis used a 5-year time horizon because it was considered adequate for recording progressive events and changes in patients’ histories without compromising the predictive power of the model [23]. Although the base case model setting was a 5-year time horizon, the flexibility available in the model permitted sensitivity analyses over a varying time horizon from 1 year to 30 years. The starting patient population for the model was 1,000 patients per treatment run for 100 replications per scenario. However, the probabilistic sensitivity analysis (PSA) encompassed 100 sets of 10 replications (also with 1,000 patients per treatment) where the relapse risks per drug and discontinuation hazards are using uniform distributions ±30%, adverse events- and relapse relative risks are using beta distributions, the costs are using gamma distributions and the relapse utilities are varied using beta distributions. The costs and projected mean LYs and QALYs are reported as discounted outcomes.

## Results

**Figure 3:**
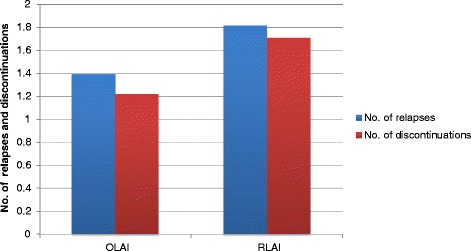
**Number of relapses and discontinuations per patient over 5 years: OLAI** ***versus*** **RLAI (base case model).**

**Figure 4:**
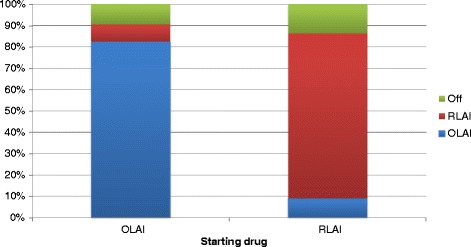
**Time on treatment: OLAI** ***versus*** **RLAI.**

The model analysis estimated OLAI to be a less costly and more effective strategy than RLAI based on all studied base-case parameters. In the base case analysis, OLAI was both cost reducing and more effective with an incremental discounted cost savings of €2,940 (SD 300.83) for gains of 0.04 (SD 0.025) LYs and 0.07 (SD 0.019) QALYs with ICER described as “dominant” for OLAI (i.e. when OLAI demonstrates cost savings and QALY gains versus RLAI it is described as “dominant” (Table 8)). Over the 5-year time horizon, OLAI treatment avoided 0.42 (SD 0.049) relapses per patient compared with RLAI. Treatment with OLAI resulted in fewer relapses and treatment discontinuations over the 5-year time horizon (Figure 3). Patients on OLAI tended to switch treatment less frequently and stayed on the treatment for approximately 82.5% (SD 0.96%) of the time compared with about 77.3% (SD 0.98%) for RLAI over the 5-year period (Figure 4). Base-case economic outcomes analysis predicts that the OLAI treatment strategy is less costly and more effective (‘dominant choice’) compared to RLAI. Thus, the model predicted that OLAI is incrementally cost-effective compared with RLAI. The ICERs did not vary greatly when using 0% and 6% discount rates compared with the base case 3% discount rate (Table 8).

**Table 8:** **Base case and sensitivity analysis results**

**OLAI** ***versus*** **RLAI**	**Incremental (discounted)**				**ICER (discounted)**		
	**Cost mean (SD)**	**LYs mean (SD)**	**QALYs mean (SD)**	**Relapses mean (SD)**	**Cost/QALY**	**Cost/relapse avoided**	**Cost/life year gained**
**Base case**	**-€2939.66 (300.83)**	**0.04 (0.025)**	**0.07 (0.019)**	**−0.42 (0.049)**	**Dominant**	**Dominant**	**Dominant**
Revised OLAI dosing schedule (2 weekly starting dose & 4 weekly maintenance dose)	-€2810.58 (281.38)	0.04 0.021)	0.07 (0.018)	−0.41 (0.046)	Dominant	Dominant	Dominant
Discount Rate set to 0%	-€3068.23 (321.06)	0.04 (0.027)	0.08 (0.020)	−0.42 (0.049)	Dominant	Dominant	Dominant
Discount rate set to 6%	-€981.51 (125.16)	0.0001 (0.000)	0.0005 (0.000)	−0.42 (0.049)	Dominant	Dominant	Dominant
Relapse rate of OLAI equal to RLAI (0.176)	-€2513.30 (286.30)	0.04 (0.024)	0.05 (0.018)	−0.34 (0.044)	Dominant	Dominant	Dominant
Discontinuation of OLAI equal to RLAI (0.445)	-€1594.46 (324.59)	−0.003 (0.027)	−0.003 (0.019)	−0.07 (0.050)	€472796.00	Dominant	€491207.00
Maximum number of switches set to 5	-€2937.57 (314.19)	0.04 (0.023)	0.07 (0.017)	−0.42 0.050)	Dominant	Dominant	Dominant
Maximum number of switches set to 10	-€2947.24 (318.27)	0.04 (0.022)	0.07 (0.017)	−0.42 (0.052)	Dominant	Dominant	Dominant
Cost of routine management^1^ increased 30%	-€3527.51 (371.13)	0.04 (0.025)	0.07 (0.019)	−0.42 (0.049)	Dominant	Dominant	Dominant
Cost of routine management^1^ decreased 30%	-€2351.82 (232.74)	0.04(0.025)	0.07 (0.019)	−0.42 (0.049)	Dominant	Dominant	Dominant
Yearly adverse event rate of OLAI equals RLAI	-€2878.91 (319.59)	0.04 (0.023)	0.09 (0.016)	−0.41 (0.052)	Dominant	Dominant	Dominant
Cost of administration increased 30%	-€3083.92 (303.46)	0.04 (0.025)	0.07 (0.019)	−0.42 (0.049)	Dominant	Dominant	Dominant
Cost of administration decreased 30%	-€2795.41 (298.37)	0.04 (0.025)	0.07 (0.019)	−0.42 (0.049)	Dominant	Dominant	Dominant
Cost of administration of OLAI equals RLAI	-€3392.17 (301.51)	0.04 (0.025)	0.07 (0.019)	−0.42 (0.049)	Dominant	Dominant	Dominant
Time Horizon 1 year	-€643.44 (116.98)	0.02 (0.004)	0.02 (0.003)	−0.11 (0.023)	Dominant	Dominant	Dominant
Time Horizon 2 years	-€1402.48 (168.63)	0.02 (0.009)	0.03 (0.007)	−0.21 (0.031)	Dominant	Dominant	Dominant
Time Horizon 3 years	-€2005.29 (224.89)	0.02 (0.015)	0.05 (0.011)	−0.29 (0.038)	Dominant	Dominant	Dominant
Time Horizon 4 years	-€2493.99 (267.06)	0.03 (0.019)	0.06 (0.014)	−0.36 (0.044)	Dominant	Dominant	Dominant
Time Horizon 10 years	-€4552.43 (513.34)	0.11 (0.057)	0.11 (0.040)	−0.72 (0.072)	Dominant	Dominant	Dominant
Time Horizon 15 years	-€5491.36 (746.04)	0.19 (0.089)	0.14 (0.059)	−0.96 (0.088)	Dominant	Dominant	Dominant
Time Horizon 20 years	-€6010.09 (985.99)	0.28 (0.118)	0.16 (0.076	−1.16 (0.111)	Dominant	Dominant	Dominant
Time Horizon 30 years	-€6184.28 (1387.21)	0.44 (0.172)	0.20 (0.104)	−1.41 (0.144)	Dominant	Dominant	Dominant
Utility tariffs increased 30%	-€2939.66 (300.83)	0.04 (0.025)	0.09 (0.024)	−0.42 (0.049)	Dominant	Dominant	Dominant
Utility tariffs decreased 30%	-€2939.66 (300.83)	0.04 (0.025)	0.05 (0.013)	−0.42 (0.049)	Dominant	Dominant	Dominant
Probability of doctors visit for adverse events 5%	-€2983.18 (269.74)	0.04 (0.023)	0.07 (0.019)	−0.43 (0.044)	Dominant	Dominant	Dominant
Probability of doctors visit for adverse events 15%	-€2824.75 (326.69)	0.04 (0.027)	0.07 (0.020)	−0.41 (0.050)	Dominant	Dominant	Dominant
Probability of switching treatment following doctors visit for adverse events 5%	-€2940.17 (303.80)	0.04 (0.024)	0.07 (0.017)	−0.42 (0.051)	Dominant	Dominant	Dominant
Probability of switching treatment following doctors visit for adverse events 25%	-€2849.86 (334.18)	0.04 (0.024)	0.07 (0.018)	−0.41 (0.054)	Dominant	Dominant	Dominant
Cost of OLAI increased 25%	-€152.19 (302.58)	0.04 (0.025)	0.07 (0.019)	−0.42 (0.049)	Dominant	Dominant	Dominant
Cost of OLAI decreased 25%	-€5727.14 (304.63)	0.04 (0.025)	0.07 (0.019)	−0.42 (0.049)	Dominant	Dominant	Dominant
Branded drug costs decreased 7.5%	-€2899.05 (296.94)	0.04 (0.025)	0.07 (0.019)	−0.42 (0.049)	Dominant	Dominant	Dominant
Cost of concomitant medication increased 25%	-€2934.53 (302.95)	0.04 (0.025)	0.07 (0.019)	−0.42 (0.049)	Dominant	Dominant	Dominant
Cost of concomitant medication decreased 25%	-€2944.80 (298.74)	0.04 (0.025)	0.07 (0.019)	−0.42 (0.049)	Dominant	Dominant	Dominant

^1^Including the variables cost of schizophrenia management, inpatient cost of relapse management, outpatient cost of relapse management.

SD = Standard deviation.

Patients who start on RLAI and later switched to OLAI in the model face the small but non-zero risk of experiencing post-injection syndrome. In the model, “switch costs” are accrued to the starting drug, which is consistent with the intention-to-treat principle. When the yearly relapse hazard for OLAI is set to equal that for RLAI, implying absence of any difference in relapse hazard between OLAI and RLAI, the ICERs for OLAI are dominant for both QALY and for relapse avoided ratios.

As the time horizon extended beyond 5 years, the model showed that OLAI increased the number of QALYs accrued, decreased the relapse rate, and tended to stabilise the discounted ICER costs per QALY and per relapse avoided (Table 8). The dominant character of OLAI persisted when additional factors were altered.

Sensitivity analyses demonstrated that OLAI remains dominant and below the acceptable threshold of €30,000 [37] incremental cost per QALY across a wide range of analyses (Table 8), demonstrating the robustness of the results. Compared with the base case, OLAI remained dominant when the utility tariffs were varied (Table 8).

As per the SPC for OLAI [14] a sensitivity analysis was also run examining the cost effectiveness of a 210 mg/2 weeks starting dosing (keeping the maintenance dose equivalent to 300 mg/4 weeks) schedule and was found to have a negligible impact on the results (see Table 8). Thus, the dosing schedule as utilised in the model was deemed representative.

**Figure 5:**
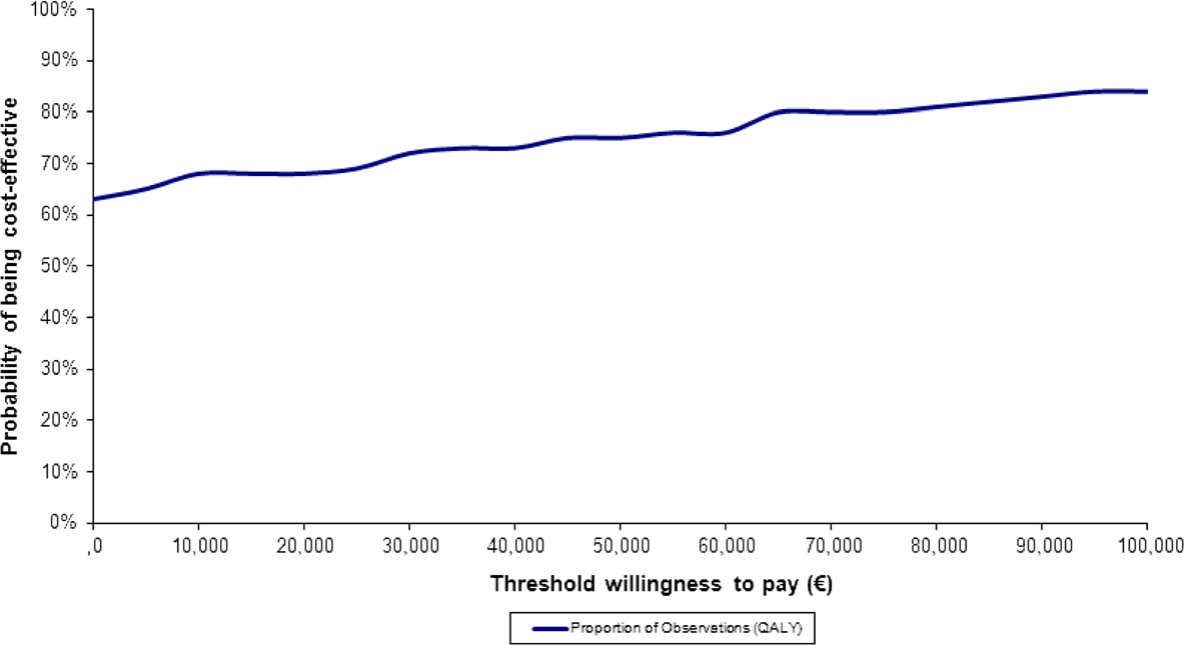
**OLAI** ***versus*** **RLAI – CEAC.**

**Figure 6:**
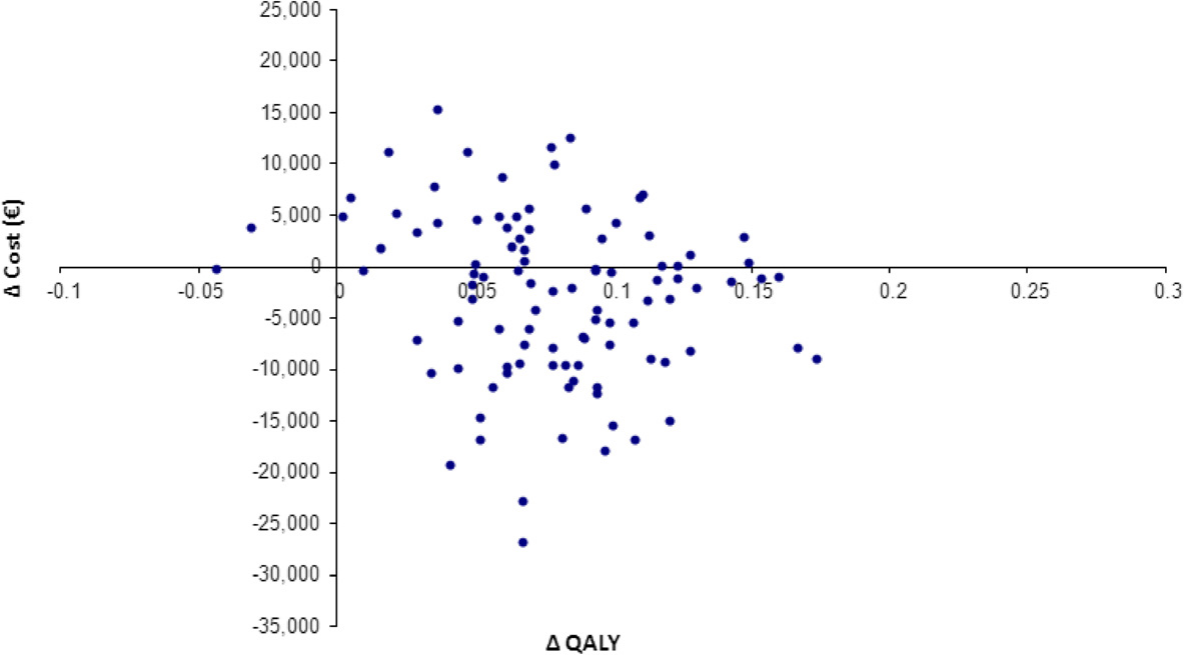
**Cost-effectiveness plane for OLAI** ***versus*** **RLAI.**

Based on the cost-effectiveness acceptability curve (CEAC), OLAI is seen as a cost-effective alternative to RLAI with a probability of 84% at a cost-effectiveness threshold of €100,000, at 80% for a threshold of €75,000, and at 72% for a €30,000 threshold (Figure 5). The cost-effectiveness plane based on net discounted benefit supports the robustness around analytical and methodological findings for the base case (Figure 6).

## Discussion

This is the first pharmacoeconomic model to compare OLAI with RLAI in the treatment of schizophrenia within the Spanish healthcare system. The DES model designed for this study found OLAI dominant within the societal willingness-to-pay threshold of €30,000 per QALY [37]. Over the 5-year period, the base case estimated that OLAI produced an additional 0.07 (SD 0.019) discounted QALYs gained and 0.04 (SD 0.025) LYs gained per patient with a dominant discounted incremental cost savings of €2,940 (SD 300.83) and a dominant ICER per QALY and per relapse avoided compared with RLAI. The treatment continuation rate was higher for OLAI versus RLAI suggesting that OLAI has a better treatment persistence rate suggesting OLAI might have the potential for a low risk of discontinuation. The main differences across the two treatments appear to be linked to the yearly hazards for relapse and discontinuation, which are lower with OLAI. Sensitivity analyses using varying discount rates of 0% and 6%, or varying model time horizons between 1 and 30 years favoured the OLAI treatment strategy. Over a time horizon of less than 5 years, OLAI had a reduced QALY advantage but was still dominant. However, as the time horizon was extended beyond the 5-year mark, there was a progressive but substantial increase in the number of QALYs accrued. Concurrently, the annual rate of relapses per patient decreased. Overall, the estimates derived by the economic model show that OLAI represents a cost-effective treatment option compared with RLAI.

The incremental cost per QALY gained with one treatment versus another is widely recognized as an acceptable payer metric of cost-effectiveness. Overall, from a cost-effectiveness perspective, OLAI was the dominant therapy in terms of cost/QALY gained, cost/LY gained, and cost/relapse averted because it was predicted to produce more QALYs at a lower cost. Factors that make OLAI an alternative and cost-effective option versus RLAI in the management of patients with schizophrenia include higher QALYs, lower yearly relapse hazard, the lesser probability of treatment discontinuation, the increase in the number of QALYs accrued, and the number of relapses averted as the time horizon was extended. Notwithstanding the model assumption of a 3-hour post-injection observation period because of the reported low incidence of post-injection syndrome, OLAI exhibits a dominant discounted ICER for cost of administration. This is because RLAI is administered twice in a month and OLAI only once per month. Additionally, RLAI therapy requires oral supplementation for the first 3 weeks of therapy [15].

This model has a number of limitations. The most important limitation concerns the lack of head to head clinical data for these two therapies. The study design of the clinical trials for OLAI and RLAI meant that an adjusted indirect comparison was not possible, so instead relapse and discontinuation rates were taken directly from the clinical trials adjusted for patient years of exposure and substantiated with expert clinical opinion.

The model calculations used available data from the literature and relied upon multiple data sources. Another limitation of the model is its exclusion of indirect cost data, which can represent a substantial proportion of the total costs for the treatment of schizophrenia [12]. Further, the model assumed the rate of hospitalisation as a proxy for relapse rates and did not account for the naturalistic fluctuations in relapse and treatment discontinuation over time.

Notwithstanding the limitations discussed above, this model has several strengths. It simulates the real world treatment processes and environment and provides projections that should help inform decision-making processes. An important strength of this model is the dynamic nature of usual care. The model simulates the real-world environment that involves switching of treatment, discontinuations, stopping/restarting treatment. The model assumptions were substantiated by clinical experts and relied upon outputs that are relevant for comparing antipsychotic drug therapy. Sensitivity analyses helped establish the validity and robustness of the model findings. This model can be applied to different time horizons up to a lifetime permitting projections that demonstrated that as the timelines were extended OLAI became increasingly cost-effective.

## Conclusions

The present analysis demonstrated that in the context of the Spanish healthcare setting OLAI was dominant compared to RLAI. The base case estimates demonstrated that the OLAI strategy resulted in an additional 0.07 (SD 0.019) QALYs and 0.04 (SD 0.025) LYs gained per patient. In addition, patients on OLAI experienced lower number of relapses and discontinuations per patient over the 5-year time horizon with cost-savings of €2,940 (SD 300.83).

The lower risks of relapse and discontinuation ensure that OLAI therapy reduces the cost of patient management, lowers the risk of rehospitalisation and associated costs, and improves the overall cost-effectiveness of OLAI with cost of therapy remaining within the acceptable threshold of €30,000 incremental cost per QALY gained. The lower risks of relapse and discontinuation ensure that OLAI therapy reduces the cost of patient management, lowers the risk of rehospitalisation and associated costs and results in OLAI being cost-effective at a threshold of €30,000 per QALY in Spain. The results support the use of OLAI therapy as an alternative to RLAI in patients with schizophrenia.

## Abbreviations

OLAI: Olanzapine long-acting injection
RLAI: Risperidone long-acting injection
DES: Discrete event simulation
LYs: Life years
QALYs: Quality-adjusted life years
SOHO: Schizophrenia Outpatient Health Outcomes
OLZ: Oral formulation
WHO: World Health Organization
SPC: Summary of Product characteristics
ICER: Incremental cost-effectiveness ratio
SCAP: Schizophrenia care and assessment programme
CI: Confidence interval
PSA: Probabilistic sensitvity analysis
CEAC: Cost-effectiveness acceptability curve
SD: Standard deviation
